# Multi-dimensionality and variability in folk classification of stingless bees (Apidae: Meliponini)

**DOI:** 10.1186/s13002-015-0029-z

**Published:** 2015-05-23

**Authors:** Fernando Zamudio, Norma I. Hilgert

**Affiliations:** Instituto Multidisciplinario de Biología Vegetal (CONICET-UNC), Av. Vélez Sársfield 1611 (X5000HVA), 5000 Córdoba, Argentina; Instituto de Biología Subtropical, CONICET, Facultad de Ciencias Forestales, Universidad Nacional de Misiones, Bertoni 85, 3370 Puerto Iguazú, Misiones Argentina

**Keywords:** Folk classifications, Local variability, Culturally mixed population, Prototypicality, Binomiality

## Abstract

**Background:**

Not long ago Eugene Hunn suggested using a combination of cognitive, linguistic, ecological and evolutionary theories in order to account for the dynamic character of ethnoecology in the study of folk classification systems. In this way he intended to question certain homogeneity in folk classifications models and deepen in the analysis and interpretation of variability in folk classifications. This paper studies how a rural culturally mixed population of the Atlantic Forest of Misiones (Argentina) classified honey-producing stingless bees according to the linguistic, cognitive and ecological dimensions of folk classification. We also analyze the socio-ecological meaning of binomialization in naming and the meaning of general local variability in the appointment of stingless bees.

**Methods:**

We used three different approaches: the classical approach developed by Brent Berlin which relies heavily on linguistic criteria, the approach developed by Eleonor Rosch which relies on psychological (cognitive) principles of categorization and finally we have captured the ecological dimension of folk classification in local narratives. For the second approximation, we developed ways of measuring the degree of prototypicality based on a total of 107 comparisons of the type “X is similar to Y” identified in personal narratives.

**Results:**

Various logical and grouping strategies coexist and were identified as: graded of lateral linkage, hierarchical and functional. Similarity judgments among folk taxa resulted in an implicit logic of classification graded according to taxa’s prototypicality. While there is a high agreement on naming stingless bees with monomial names, a considerable number of underrepresented binomial names and lack of names were observed. Two possible explanations about reported local naming variability are presented.

**Conclusions:**

We support the multidimensionality of folk classification systems. This confirms the specificity of local classification systems but also reflects the use of grouping strategies and mechanisms commonly observed in other cultural groups, such as the use of similarity judgments between more or less prototypical organisms. Also we support the idea that alternative naming results from a process of fragmentation of knowledge or incomplete transmission of knowledge. These processes lean on the facts that culturally based knowledge, on the one hand, and biologic knowledge of nature on the other, can be acquired through different learning pathways.

## Background

The ability to classify nature discontinuities is an innate trait of human beings in which cognitive, psychological, symbolic, utilitarian and even cosmological aspects interact [1–5]. Categorization is guided by a first basic principle of cognitive economy that maximizes information with the least cognitive effort and a second principle that supposes the perceived world comes as structured information rather than as arbitrary or unpredictable attributes [6]. In turn, category systems have vertical and horizontal dimensions; the first concerns the level of inclusiveness of the category and the second the segmentation of categories at the same level of inclusiveness.

Contrary to the notion of categories as boxes which include or exclude items based on necessary and sufficient conditions like scientific taxonomies, Eleanor H. Rosch and colleagues [6, 7] conceive natural categories as graded structures without clear-cut boundaries. In this, process similarity plays a fundamental role, and thus, serves as an organising principle by which individuals classify objects, form concepts, and make generalizations [8]. But similarity judgments, regarded as extensions of similarity statements, are asymmetrical, given the relative salience of objects. In a statement of the form “a is like b” people tend to select the more salient stimulus, or the prototype, as a referent, and the less salient stimulus, or the variant, as a subject [6, 8]. These facts led Rosch and colleagues [7] to propose which categories are graded in terms of their clear cases (prototypes) rather than of their boundaries. For these researchers, perception of typicality differences (or prototypicality) is, in the first place, an empirical trait of people’s judgments about category membership.

Despite the Prototype Theory proposes a convincing explanation of graded organization (see [9]) and prototypicality is a recurrent fact in folk classifications, this theory has only been marginally referred to in ethnosciences studies. This has been due in part to the predominant use of some theoretical and methodological approaches (e.g. linguistic) which do not allow to capture other dimensions of the folkloric classification systems. In this sense, Hunn [3] addressed ethnobiologists to change the study classification systems in use towards a combination of cognitive, linguistic, ecological and evolutionary theories in order to account for the dynamic character of ethnoecology.

Also the apparent contradiction between the singularity and universality of folk classification has been partially overcome so as to recognize the coexistence of two broad classification systems [10]; a hierarchical general-purpose classification based principally on morphological mechanisms [5, 10–13] and a non-hierarchical special-purpose classification based on ecological, symbolic and other mechanisms [1, 14–17]. Hence, folk classifications began to be referred in the literature as multidimensional systems or multi-mechanistic taxonomies [4, 18].

However, beyond the particularities and the inherent variability observed within ethnic groups, certain homogeneity of the proposed general models in folk classifications should be taken with caution [19]. While local variability in taxonomic assignment is well documented [10, 19, 20], analysis and interpretation of variability in folk classifications have usually played a secondary role.

The structure and meaning of nomenclatures have been central to the discussions developed around folk classifications [20–25]. They have not only allowed the elicitation of the structure of classifications, but also enabled us to explore hypotheses about socio-ecological processes [26, 27]. For example, the meaning of monomial and binomial names has allowed the discussion on the universalism and utilitarian conceptions of folk classifications [22], and on the relationships among organisms characteristics (e.g. salience) and their role in culture [22, 24].

Moreover, the overall agreement among members of a cultural group on names of one domain of knowledge allows us to propose hypotheses and explanations about the origin of cognitive diversity [28]. Polysemy and other naming variations -including the absence of names or lack of knowledge about names- can give clues to the knowledge pattern and learning processes of individuals in a particular location. While there may be an unresolved dispute about the existence of “culturally correct names”, in any case the “inconsistency in naming reflects the informant’s uncertainty” [28]. On the contrary, Boster [28] and Romney et al. [29] argue that the more an informant agreed with others the more cultural knowledge that informant had about any domain.

A further consideration was made by Gardner [19] who noted that meanings of folk nomenclatures can change over people’s lifetime as a result of inductive processes, experimentation, and/or feedback with the environment.

The aim of this paper is to determine how rural residents of the Atlantic Forest of Misiones (Argentina) classify honey-producing stingless bees (hereafter SBs) according to the linguistic, cognitive and ecological dimensions of the folk classification system. Through these comparative analysis we seek to identify similarities and differences between the different dimensions of the system of classifications studied, as well as to identify the psychological mechanisms and grouping strategies used. We used the “grouping strategies” concept to refer to the spatial representation or “figure” resulting from judgment obtained from membership categorization performed by people (e.g. hierarchical) and not to indicate the distance or degree of closeness between items of one domain listed sequentially (See [30]). Finally we analyzed the socio-ecological meaning of binomiality in naming and the meaning of local variability in the appointment of SBs.

## Methods

### Studied area and population

**Fig. 1:**
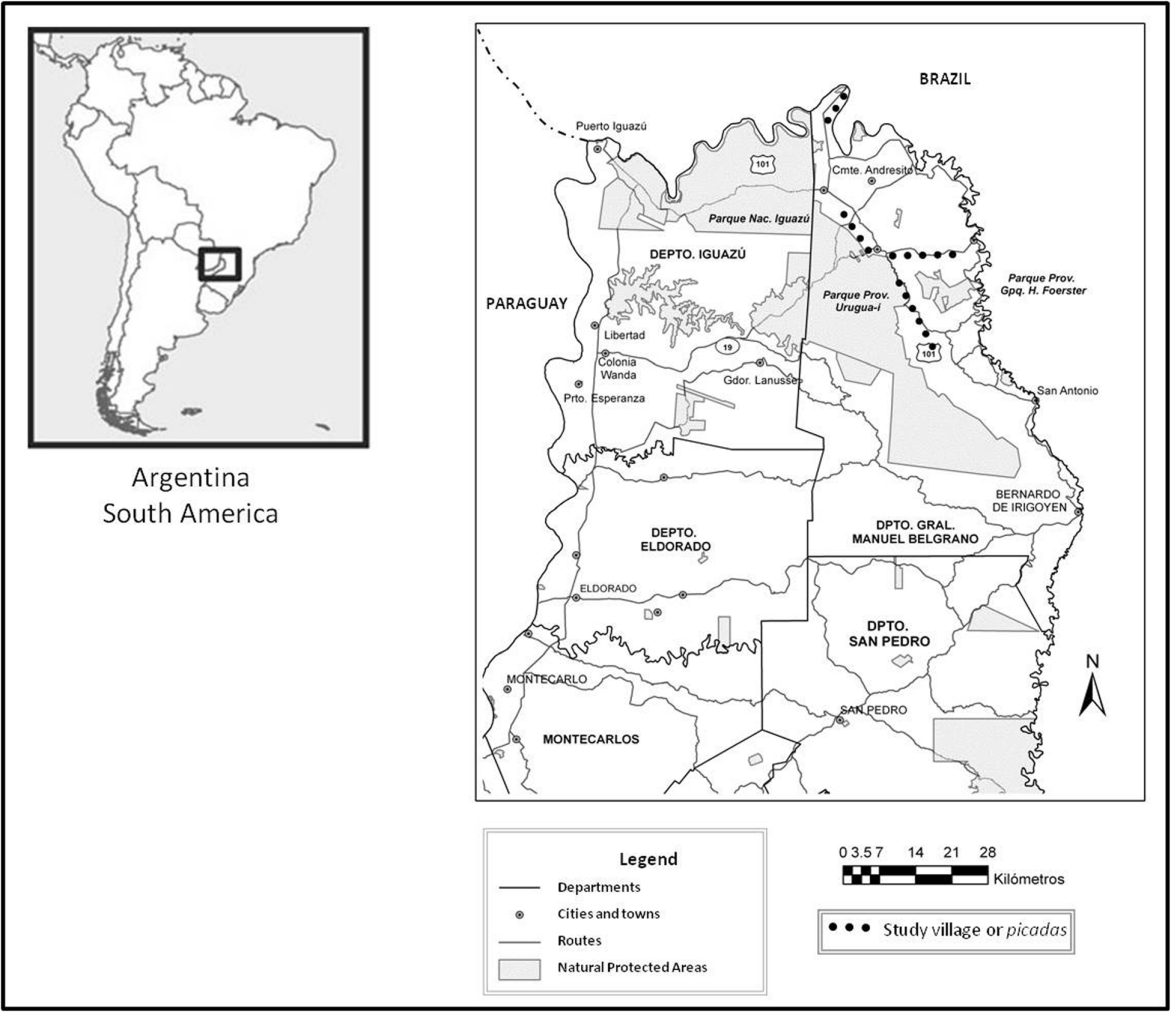
Map of the study region and *picadas* where interviews were conducted

Research was conducted in the northern part of Misiones province (Argentina) in the department of General Manuel Belgrano on the northern and northeastern border with Brasil (Fig. 1). Study areas are within the Atlantic Forest Ecoregion [31]. This is a semi-deciduous forest growing in a subtropical climate (1700-2200 mm annual rainfall) with hot summers (35-40 °C) [32].

The economy in the area is based on raw material extraction with little industrial development [33]. The population of Misiones province results from the conjunction and coexistence of the original indigenous population (Mby’a-Guaraní), European and Asiatic immigrants that arrived between 1900 and 1940, and Paraguayan and Brasilian families that fled to the province during the twentieth century. To these farmers of mixed cultural background who live alongside each other in a culturally mixed population, we will call *Criollos* in order to differentiate them from other people who live in culturally homogeneous towns as Wanda where Polish descendants predominate. The *Criollos* are part of a heterogeneous local culture characterized by a strong Brazilian cultural influence; therefore, they alternatively speak Spanish and Portuguese (see [34]).

The rural population of the north of Misiones is spatially and culturally shaped by the recent history of colonization. It is one of the last colonized areas in the province and the country (~1980). It is characterized by precariousness in the legal possession of the land, an anarchic allocation of holdings and poor infrastructure development (see [33]). We have worked in different villages called *picadas* in three rural areas (Fig. 1). All farmers interviewed are small producers (5 a 50 ha) who combine commercial cultivation of tobacco or *yerba mate* (*Ilex paraguariensis*) with small agricultural plots for subsistence, supplemented by raising farm animals.

### Research design

Fieldwork was conducted between July 2007 and December 2009. Sixty-eight rural residents 16 - 79 years old were interviewed (5 aged 16- 26; 11 aged 27- 37; 8 aged 38-48; 22 aged 49-59; 14 aged 60-70; 4 aged 71-81). Not to bias the sample selection and in order to include the local heterogeneity -in terms of degree of expertise and knowledge of the informants- the selection of the informants combined random sampling with the snowball technique [35]. Both forms led to both non-specialist (n = 60) and specialist (n = 8) informants defined here as those who knew < 9 and > 9 folk taxa respectively. Most interviewees were male (4 women and 64 men), as men are those who are continually in contact with forest areas (greater chance of encountering bees) unlike women who engage in domestic and productive activities around the house.

Interviews were semi-structured and were based on initially free interviews that developed during a first visit to informants [35]. Free listings were also used in order to understand the structure and scope of the studied domain [35, 36]. To do this, we requested respondents to name all “bees that do not sting and give honey” they knew. Different topics were addressed during the interviews: folk names, criteria and descriptors used locally to describe bees, organoleptic characteristics of honey, local ecological knowledge, and the use and management of stingless bees. Additionally, specific visits were conducted to collect information on the informant’s life stories. All interviews, which took between 30 and 45 min each, were performed by FZ. Most interviews were conducted several times and in different contexts with the same informant.

A significant effort was made to ensure an entomological collection representative of SB diversity in the region. To achieve this we performed runs with informants to locate bee colonies they had previously identified. We also built our own collection of bees in systematic samplings with different collecting methods (hand nets, bait traps and pan traps). These collections were made in both rural areas and forests near protected areas (Iguazú National Park, Provincial Reserve Urugua-í and Private Reserve Urugua-í).

The specimens were properly conditioned and were deposited in the entomological collection of the research group in Ethnobiology of Subtropical Biology Institute (IBS-UNAM). Most of the collected SBs were determined by specialists (Ferando A. Silveira, Claus Rasmussen) while the remaining specimens were determined to genus level by FZ and local taxonomist Leopoldo Alvarez using Meliponini genera keys [37] and through other sources like specimens of La Plata Museum (UNLPyM). Except for the *mandasaia* (*Melipona quadrifasciata*) specimens all other folk taxa were collected. The resulting collection is the newest and most complete for Meliponini bees in the Atlantic Forest in Argentina (Leopoldo Alvarez pers. comm.).

Data from interviews were loaded into a database (spreadsheet) where folk taxa were related to the topics dealt with during the interviews. Folk names were collated with the biological characteristics mentioned in descriptions carried out by interviewees and with the SB specimens collected throughout the study. Thus, we were able to identify the biological species described and capture the variations in their naming; that is, variations in pronunciation, synonyms and cognate names. “Single mention” names describe species whose names are not known, other apparently anomalous names were also retained to analyze the meaning of the information that deviates from general agreement.

### Data analysis

To analyze the linguistic, cognitive and ecological dimensions of folk classification of SBs (horizontal dimensions of categorization systems), we used different theoretical and methodological approaches. On the one hand, we used approaches developed by Brent Berlin [38] which rely heavily on linguistic criteria. On the other hand, we used approaches developed by Rosch and colleagues [7] which rely on psychological (cognitive) principles of categorization. For the analysis of the aforementioned approaches, we used the data obtained from all the informants. Finally we captured the ecological dimensions of folk classification in narratives documented along field work but in particular in interviews with a single key informant.

In the first approach we analyzed the structure and semantics of folk names according to the structure of monomial (primary and complex primary names) or binomial names (secondary names) and we identified monotypic and polytypic generic folk taxa. Following Berlin [38], generic monotypic are composed of a single specific taxon and polytypic generic are composed of more than one folk specific taxon or folk varieties. As we worked with a particular bound domain -“bees that do not sting and give honey”- no information is provided on the higher inclusion levels in which this group is conceptually situated (vertical dimension of categorization systems) from the local perspective, such as *unique beginner* and *life forms*. To represent the overall structure of the domain we used Venn diagrams under the conventions used by Berlin [38] with minor variations and tables where other type of information is listed (e.g. folk names, scientific names, synonyms, contrasts criteria used, agreement among informants). We use the term “agreement” (high, low, higher or lower), to refer qualitatively to the consistency of the data on the appointment of bees, taking as reference the number of mentions each folk name received. In some cases percentages were calculated, but the nature of data did not justified statistical analysis.

In the second approach we used spontaneous comparisons of the type “folk taxon X is similar to ethnotaxon Y” exposed by informants during interviews, to describe how bees are like and how they behave. In this way we broach the study considering Rosch [6], who proposed that natural languages possess linguistic coding and mechanisms for coping with gradients of category membership. The latter and Tversky [8] show that in sentence frames like those spontaneously given by our informants, the more prototypical member of a pair of items is placed into the referent slot (folk taxon Y in the example above) even under changes of both instructions and item, in experimental tests (see [39]). According to Tversky [8] “proximity data from both comparative and production tasks reveal significant and systematic asymmetries whose direction is determined by the relative salience of the stimuli”.

Based on a total of 107 comparisons of the type “X is similar to Y” identified in personal narratives, where X is the folk taxon described and Y the referent folk taxon, we performed a matrix of two entries. Then we applied a Principal Component Analysis (PCA) which provides a graphic representation of the folk taxa according to the similarity between them (correlation matrix) and according to the folk taxa that acted as a referent to describe the other. To perform the PCA we used the Infostat program [40].

Also we calculated the Prototype Value (PV) of folk taxa according to the following formula: SV = a/[(b + c) ‐ 1], where

- a = Number of times X was used as referent folk taxon.
- b = Number of folk taxa that was used to describe X folk taxon.
- c = Total number of comparisons used to describe X folk taxon.

We consider that the folk taxa more frequently used as referent to describe others will have a higher value of prototypicality, which agrees with the idea that there are no prototypical organisms but organisms with different degrees of prototypicality [6]. In turn, this value decreases while the number of folk taxa (b) and citations (c) that are used to describe another folk taxon increase. *Apis mellifera* (hereafter *abeja*) was also included in both analyzes (PCA and PV), because it was repeatedly used by informants (18) to describe some folk taxa of SB. However, we do not inquire about the opposite, ie which taxa are describing to the *abeja*. Therefore, given that *abeja* is a common and easily distinguishable species, we assumed that one (1) folk taxon and only one (1) comparison is required to describe *abeja.* These reference values were obtained from *yateí*; this is the most salient folk taxon of SB group in the study region and also the most used as medicinal honey along with *abeja’s* honey [34, 41, 42].

Finally we analyzed the ecological grouping and discrimination of SBs provided by a single key specialist (B.L. 50 years old). This case stood out from the rest of the informants, so it was considered appropriate to show the nature of the folk classifications in all its dimensions. But beyond his knowledge and skills (similar to that of other specialists), we want to analyze and highlight the contributions he made particularly in one of the interviews (23/01/08, San Antonio, recording No. 2). In this interview B.L. spontaneously argued his own way of understanding the “bees that do not sting and give honey”, giving us information about a unique functional classification system. B. L. knows the diversity and biology of SBs in detail and has a predilection for honey. He is one of the few informants who continues to practice the harvesting of wild honey and still maintains a close relationship with forest areas. This is due to a singular personal history (his father was a pioneer in the region) and to the fact that he has lived since his early childhood at the edge of Urugua-í Provincial Park, which preserves 84,000 hectares of Atlantic Forest (Fig. 1).

## Results

We found a total of 50 SB names excluding language variations, synonyms and cognate names. Among these, 45 % received a single mention, 30 % between two and 10 mentions and 25 % over 11 mentions. Additionally, in 34 occasions the bees were described by the majority of informants but they did not know their names (hereafter “unnamed bees”). In 12 occasions there were inconsistencies between the description of the species and the given folk name if we consider the rest of the informants (hereafter “exceptions”) (Table 1).

**Table 1:** Stingless bees general purpose classification. The table shows the identity of generic folk taxa (1st column), number (2nd column) and the identity of specific folk taxa (3rd column), and characteristic contrasts used by the *Criollos* for grouping and differentiating etnotaxa (4th column). See an example of how to interpret the table, in footnotes

Generic ethnotaxa			# Specific ethnotaxa	Specific ethnotaxa		Contrasts	
Folk names	Specie	Synonyms		Folk names	Specie	Grouping	Differentiate
*carabozá* (22)	*Trigona spinipies* [1:1]	*irapuá* (20), *carabozá negro* (3) *irapuá negro* (2)	2-3 (4-3)	*carabozá amarillo* (3)	*Tetragona clavipes*	Aggressiveness	Colour/nesting substrate
				*carabozá marrón* (1)			
		*corta pelo* (3)					
				*cabichui amarillo* (1)			
		*cabichui* (1)					
				*carabozá de madera* (1)	*Scaptotrigona* spp.		
		*abeklack* ^b^ (1)					
*iratín* (17)	*Lestrimelitta* spp. [1:2 o 3]	*veintecinco puertas* (2)	2 (1)	*iratín amarillo* (1)	*Tetragona clavipes*	Group generalities	Colour
		*sesenta puertas* (1)					
		*abeja limón* (1)					
*mandurí* (21)	*Melipona torrida* [1:1]	*mandurí de madera* (6)	2-3 (5-1)	*manduri de tierra* (1)	*Schwarziana cuadripunctata*	Group generalities	Colour/nesting substrate
				*mandurí amarillo* (1)	*Tetragona clavipes*		
			1 Var. (1)	*mandurí de madera grande* (1)	*Melipona torrida*	Body shape/color/behavior	Size
				*mandurí de madera chico* (1)			
*mirí* (38)	*Plebeia* spp. [1: 2 o 3]	*mirín* (1) *miní* (1)	2 (4)	*mirí guazú* (1)	*Nannotrigona* sp.	Group generalities/size^a^	Size
				*mirí de tierra* (1)	*Schwarziana cuadripunctata*	Group generalities	Nesting substrate
			2-3 Var (9- 3)	*mirí chico* (4)	*Plebeia* spp.	Size/body shape/general behavior	Size/Nesting substrate
				*mirí guazú* (4)			
				*mirí de piedra* (1)			
				*mirí de madera* (1)			
*tobuna* (10)	*Scaptotrigona* spp. [1: 2]	*tapezuá* (3)	2 (1)	*tobuna amarilla*	*Tetragona clavipes*	Aggressiveness	Colour
		*tobuna negra* (1)					
		*culo de burro* (1)					
		*pao de boi* (1)					
*yateí* (67)	*Tetragonisca fiebrigi* [1:1]	*goldbiene* ^b^ (1)	2 (6)	*yateí guazú* (2), *yateison* (4),	*Tetragona clavipes*	Body shape/colour	Size
			2-3 Var (12-1)	*yateí chico*, *yateí grande*, *yateí negro*, *yateí amarillo*	*Tetragonisca fiebrigi*	Size/body shape/general behavior	Size/Colour
*cagafuego* (4)	*Oxytrigona tataira* ^c^ [1:1]	-				Behavior (sting types)	Colour/Size/general morphology
*borá* (20)	*Tetragona clavipes* [1:1]	*yateison* or *yateí guazú* (6)	-	-	-	-	-
		*ebora* (2)					
*abeja del suelo* (15)	*Schwarziana cuadripunctata* [1:1]	*guira* (5), *uruzú* (4)	-	-	-	-	-
*mandasaia* (28)	*Melipona quadrifasciata* [1:1]	-	-	-	-	-	-
*mambuca* (13)	*Cephalotrigona capitata* [1:1]	-	-	-	-	-	-
*guaraipo* (20)	*Melipona bicolor* [1:1]	-	-	-	-	-	-

E.g. in the first row *carabozá* is a folk name with greater consistency to refer to *Trigona spinipies* (22 citations, # quotes in brackets). So for the majority of respondents it is fitted within generic monotypic folk taxa (1st column) and presents a 1:1 correspondence with the formal academic taxonomy [in brackets]. Furthermore *carabozá* was considered polytypic with two and three specific folk taxa (2nd and 3rd column) for four and three informants respectively (# of citations in parenthesis). The specific folk taxa were grouped by aggressiveness and were differentiated by color and nesting substrate

^a^ *mirí* comes from the Guaraní lexeme *miní* which means small in contrast to *guazú* (large). Hence *mirí guazú* makes reference to the largest in the group of small SBs. ^b^Names of German origin assigned by descendants of this nationality. ^c^ *cagafuego* name is also used in the area to name some bees of Halictidae family who “urinate” people causing skin irritation

The highest number of names with a single mention (high variability) is greater in some species such as *T. clavipes* and *Scaptotrigona* spp., with 12 and 6 names respectively (Table 1). In these cases, respondents described the bees according to the majority of informants but they called them with alternative underrepresented names (hereafter “own creation”) which mostly correspond to secondary or binomial names (Table 2). They comprise 44 % of all names, followed by simple primary names with 34 % and complex primary with 22 % (both monomials). However if we consider the frequency of use, simple primary names are the most relevant with more than 80 % of the entries (Table 2). The high frequency of these names corresponds to a domain composed almost entirely by generic monotypic folk taxa.

**Table 2:** Structure and semantic of SB folk names

Structure	N° names	Percent	Frequency	Percent
Simple primary (monomials)	17	34.0	303	80.8
Complex primary (monomials)	11	22.0	30	8
Binomial secondary	22	44.0	42	11.2
TOTAL	50	100	375	100

We show three representations of the folk classification of SBs according to the linguistic, cognitive and ecological dimensions of the domain: a hierarchical general-purpose classification, a graded classification of lateral linkage and finally, a functional special-purpose classification. We describe some characteristics of each one below.

### Hierarchical general-purpose classification

In the local terminology, Meliponini bees do not have a local name that groups them. Informants differentiated SBs from other bees mainly because of their lack of sting (although some “bite”) and because they show a different “way of life” compared to the referent *abeja*. While *abeja* deposited honey in *favos* (honeycomb), SBs do so in rounded *cantaros* or *botija*s of varying sizes (pot-honey).

**Fig. 2:**
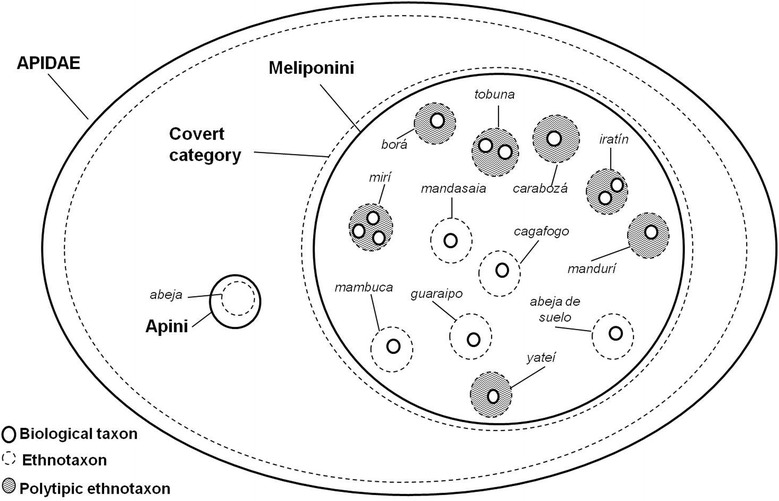
Stingless bees’ hierarchical general-purpose classifications according *Criollos* of Misiones. In the Venn diagram, it can be seen the correspondence between monotypic and polytypic taxa with biological taxa (family, tribe, and species)

Evoking “bees that do not sting and give honey” in free listings, people mentioned bees belonging to the tribe Meliponini without exception. Sometimes the word honey drove the informant towards other honey-producer bees or wasps (bumblebees of genus *Bombus* called *mamangabas* and wasps of genus *Brachygastra* and *Polybia* known as *lechiguanas* or *comatí*) but when we insisted on getting to know insects that do not sting, these folk taxa were deleted from the group mentioned. Bumblebees, wasps and *abeja* are outside the covert category composed of Meliponini bees although all of them produce honey and are close to SB as some interviewees pointed out (Fig. 2).

The domain of SB consists of 12 generic monotypic folk taxa (Fig. 2). However, six of these folk taxa were reported by at least one person as polytypic (Fig. 2 and Table 1) according to the variability in the appointment of names aforementioned. Monotypic generic folk taxa were cited 356 times (93 %) as opposed to polytypic generic, in which only 25 cases (7 %) were cited. In eight cases monotypic folk taxa are presented in a 1:1 ratio with respect to the scientific taxonomy (Table 1); while the generic folk taxa *tobuna*, *iratín* and *mirí* are made up of 2 or 3 biological species.

Except from the *mirí* folk taxa, that can refer to different species of the genus *Plebeia* or *Nannontrigona*, the folk taxa *tobuna* and *iratín* correspond to biological species of the same genus, *Lestrimelitta* and *Scaptotrigona,* respectively. *Yateí* (*Tetragonisca fiebrigi*) has a 1:1 taxonomic correspondence although people recognized between two and three ethnovarieties. Therefore, we noted that *mirí* is a sub-differentiated folk taxon and *yateí* an over-differentiated one. The generic *abeja de suelo* (ground bee) folk taxon may correspond to more than one species according to descriptions performed by informants. However, in this study we only collected specimens of *Schwarziana cuadripunctata*.

As noted above, the generic polytypic folk taxa are numerous but they were infrequently used in the studied domain. Among the polytypic generics, the most cited (83 % of cases) have at least two specific folk taxa (e.g. *carabozá* and *carabozá amarillo *-yellow *carabozá*-, Table 1, first row) while only two generic folk taxa include 3 specific (13 % of cases).

Moreover, *carabozá*, *mandurí*, *mirí* and *yateí* are polytypic folk taxa with greater agreement. People recognized two or three folk varieties of *mirí* and *yateí* folk taxa (Table 1). Folk varieties are considered variations of a specific folk taxon and were identified according to expressions such as “there are various kinds” or “there are different types”.

The structures of generic folk taxa are characterized by the group of two to three different biological species that can share implicit general characteristics common to all bees of the SB group (e.g. lifestyle features or not sting) or share some specific traits of group members (e.g. aggressive behavior, color, body shape or morphology). In both cases these characteristics may or may not be explicit in the nomenclature or be recognisable at first glance. In general, the specific folk taxa are distinguished by interviewees through contrasts between attributes related to color (nine cases), size (eight cases), nest habits (eight cases) and behavior (one case) (Table 1).

The *Criollos* used different logic to group and distinguish folk taxa. For example the generic *iratín* formed by the specific *iratín* and *iratín amarillo* do not share common features when comparing their corresponding biological species (Table 1, second row). These bees differ in their behavior, the characteristic of their *piqueras* and their body color. *Tetragona clavipes* (*iratín amarillo*) is aggressive, does not build *piquera* and individuals are yellow, while *Lestrimelitta* spp. (*iratín*) is not aggressive, builds a large tubular *piquera* and is black. These specific folk taxa were considered under the same generic because they share some or a combination of several of the implied general characteristics of the group and therefore, were considered as belonging to the “same family”. Moreover, within the generic *carabozá,* three species of different genera with a common biological aggressive behavior (Table 1) were pooled. These are differentiated by color and nesting substrate; *Trigona spinipes* is black as *Scaptotrigona* spp. and both differ from *T. clavipes,* which is yellow, while *T. spinipies* is the only species with external nest.

### Graded classification of lateral linkage

**Fig. 3:**
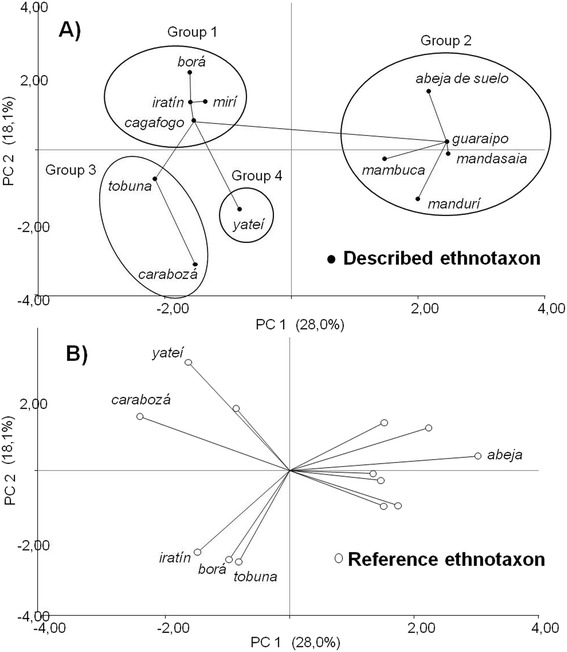
Principal Component Analysis based on similarity judgments between folk taxa made by *Criollos*. The first three components of PCA explain the 61 % of variation accumulated. For better visualization shown separately; **a**) the similarity between described folk taxa (black dots) and **b**) vectors of referent folk taxa (empty dots). The minimum spanning trees is represented by continues lines between dots. See text for description of groups (1, 2, 3, 4) in the graphic

*Yateí* and *carabozá,* on the one hand, and the *abeja,* on the other, are the referent folk taxa used to describe the rest of folk taxa according to the results of the principal component analysis (PCA) (Fig. 3b). That is, they were used to describe most of the other folk taxa while very few folk taxa were used to describe them. Thus, PC1 axis (28 % of the variability) can be interpreted as one ordering the SB domain according to the size and behavior of bees. Both characteristics are important in distinguishing the four groups of species observed (groups in Fig. 3a). In turn, other bees resembling *yateí* and *carabozá* were distinguished from those similar to *iratín*, *borá* and *tobuna*. Hence PC2 (18 % of the variability) could be interpreted as an axis explaining the value of these two prototypical folk taxa with high prototipycality. That is, they are conceptually distant from the rest of the folk taxa as well as between them.

Group 1 consists of the folk taxa that were described according to their resemblance to *yateí*, whereas *cagafuego* and *tobuna* are similar to *carabozá* (Fig. 3a). Group 2, with the *abeja* as referent folk taxon, is the most consistent since the same folk taxa were used to describe each other. Because of the morphological similarities with the larger SB subgroup, Group 2 is composed of large, robust bees, with abdominal stripes (except *mambuca*) as *A. mellifera*. Furthermore, all folk taxa within Group 2 of SB have the same evasive behavior, which enhances their closeness. In group 3 and 4 are located folk taxa that are similar to *iratín*, *borá* and *tobuna*, although there is an apparent relationship between the groups, the real distance between the points is low, as shown by the minimum spanning trees. *Carabozá* has the same color as *tobuna* and *iratín*, and shares its aggressive behavior with *borá* and *tobuna*. In contrast, *yateí* was only in a few occasions described by morphological resemblance to *borá*, while *borá* was described by its resemblance to *yateí* in many occasions (see group 1).

PCA analysis and representation are consistent with the prototypical values (PV) obtained: *yateí* is the folk taxon with highest prototypical value (0.55), followed by the *abeja* (0.34) and *carabozá* (0.05), whereas the rest of the folk taxa have much lower values (0.01-0.001).

### Functional special- purpose classification

**Fig. 4:**
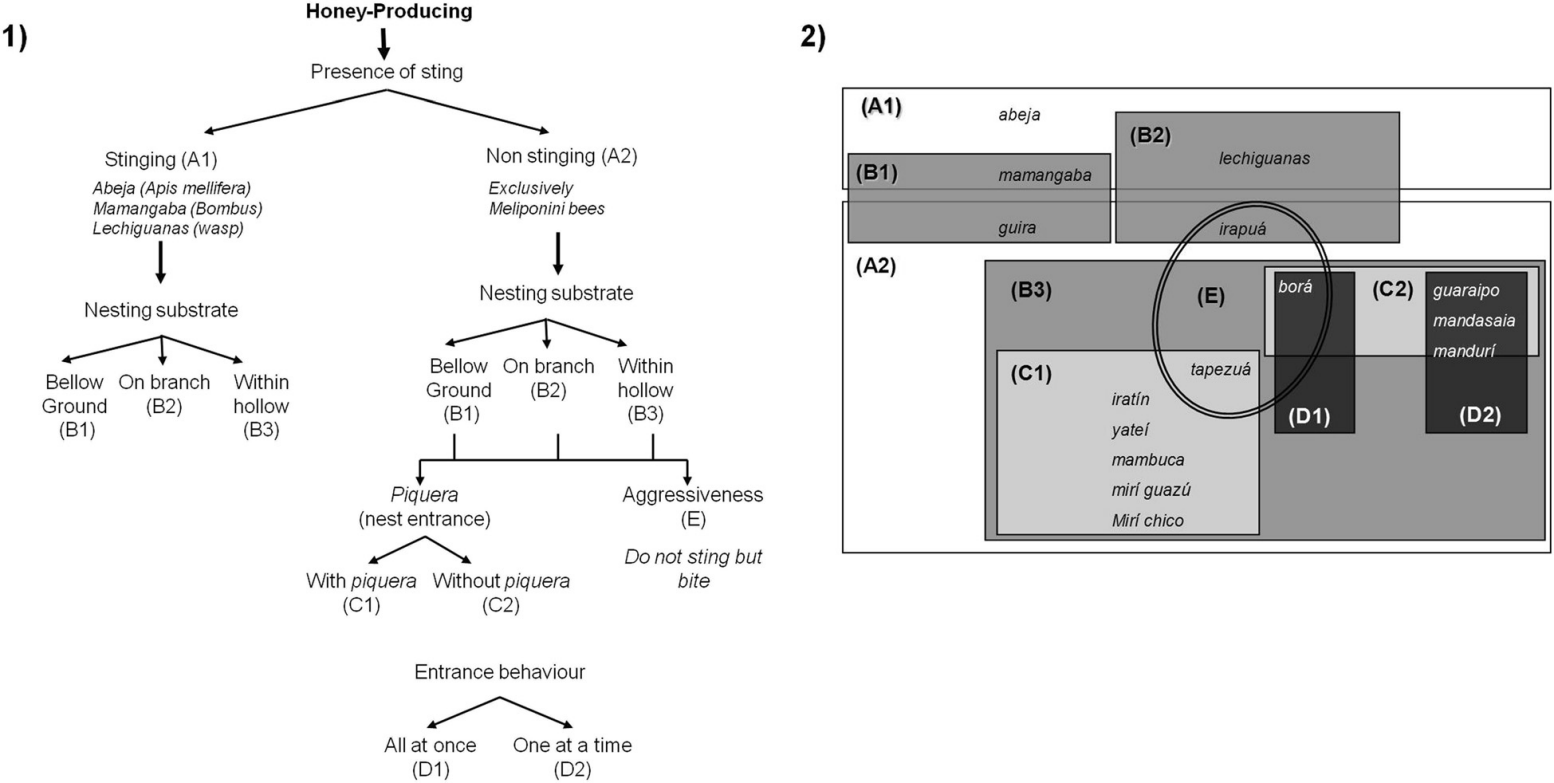
Functional special-purpose classification of the “honey producing bees” according to key informant, B.L.: (**1**) grouping criteria and contrasts used by the informant; (**2**) grouping structure of the classification system. See text for full description

The key informant B.L. explained the similarities and differences between folk taxa known to him through the use of contrasts between many different characters and criteria, as shown in Fig. 4. Although our initial question was referred to “bees that do not sting and give honey” he firstly formed a group according to their ability to produce honey as a common element of his pre-existing domain (Fig. 4). In such domain he included all known SBs and the *abeja*, and also the *mamangabas (bumblebees)*, *lechiguanas* and *comatí* wasps (paper wasps). He provided biological and morphological information for all folk taxa and also on the quality of their honeys.

He divided all bees according to the ability to sting, distinguishing bees “that sting and do not sting” (Fig. 4, A1-A2). Then he created another grouping criteria based on substrate or location of colonies, separating them in three groups (Fig. 4, B1-B3): those making nests bellow ground or “ground bees”, those making nests within tree hollows or “hollow bees”, and those making nests on tree branches. Within the group of bees that do not sting (A2) and make their colonies in tree hollows (B3), he divided bees in two new groups according to the presence or absence of *piqueras* (Fig. 4, C1-C2). Finally, within the group of bees without *piqueras*, he formed two new groups defined by the behavior of bees when entering the nest. He distinguished bees that “enter one at a time” with narrow entrance hollows and those that “enter all at once” with wide entrance hollows (Fig. 4, D1-D2). The group that enters “one at a time” included bees with evasive behavior, which leave or enter the nest intermittently and stop entering the nest when they detect people and other potential predators. On the contrary, bees that enter “all at once” can be seen at a distance, as they keep a steady flow of individuals entering and leaving the nest. Finally within the group of bees that do not sting, B.L. distinguished a group of aggressive bees that attack their enemies without a sting, called bees that “bite but do not sting” (Fig. 4, E).

## Discussion

The vernacular names collected in our region are the same as those commonly used in southern Brasil although in a few cases, they are not used to name the same biological species [43, 44]. If we consider the historical migration dynamics between Argentina and Brasil [45], this particular scenario helps, at first, to explain the confluence of new and unstable names, for us called “own creations”, and stabilized names with high agreement, possibly created a long time ago, even in other regions.

**Fig. 5:**
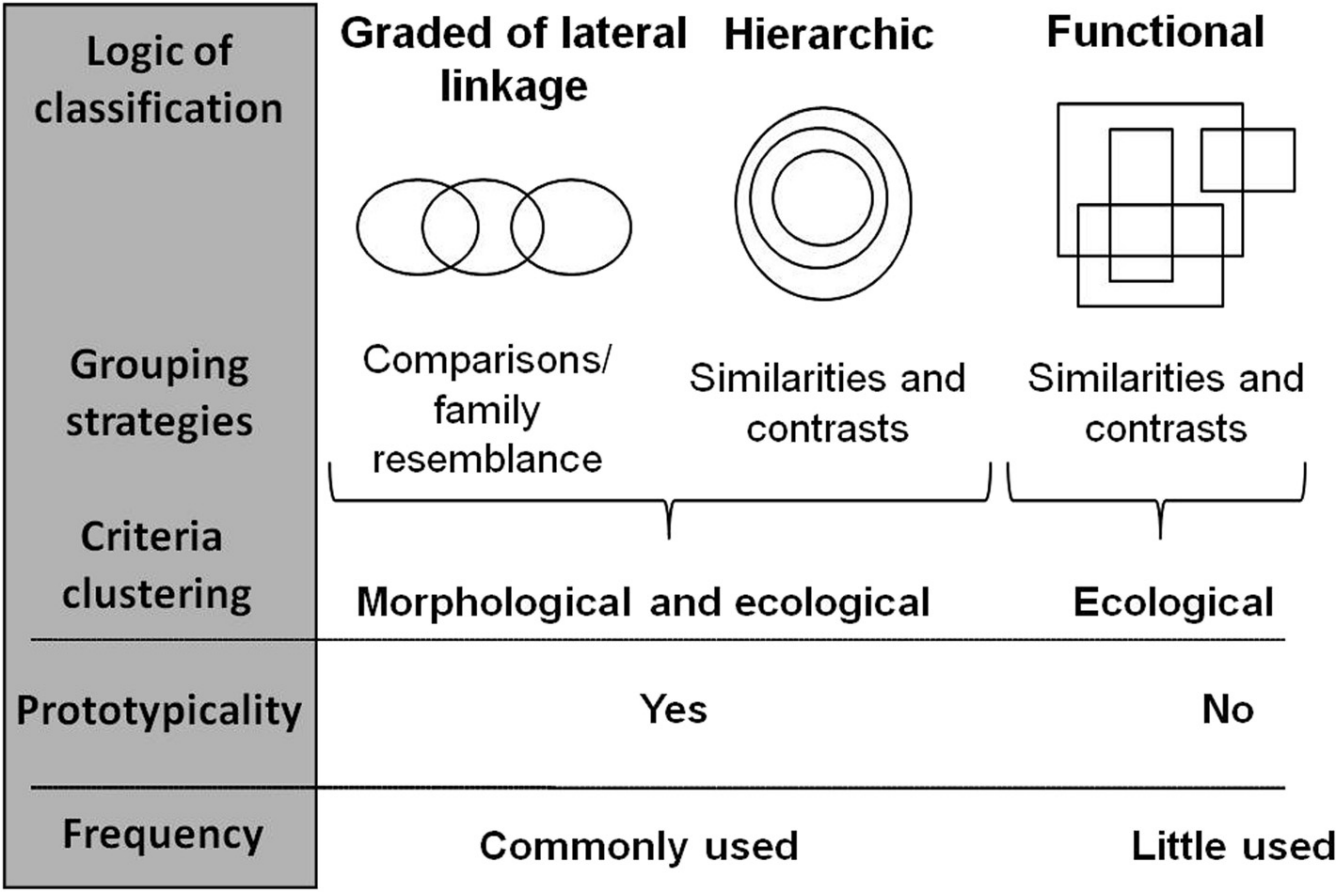
Mechanisms and structure of different classification systems of SBs identified between *Criollos* of Misiones

For the *Criollos* of Misiones, SBs are a covert category bringing together folk taxa that produce honey and do not sting. This category is ordered by the local people through different logical and grouping strategies. This leads to a hierarchical general purpose classification, which corresponds 1:1 with bees of the tribe Meliponini according to formal taxonomy, a graded classification of lateral linkage between folk taxa with different prototypicality and other functional special-purpose classifications (Fig. 5).

These three dimensions of the folk classification system coexist and are expressed according to the purpose and context in which they occur. Thereby, if the purpose is to explain the domain, lateral linkage strategies (which are informative and easy to develop) and overlapping hierarchical strategies (as in the functional classification system) are mainly used. However, if the purpose is to order the domain, a hierarchical system of general purpose with non-overlapping categories is used (Fig. 5). The hierarchical and graded classification of lateral linkage is also widely used.

Similarity judgments made in the form of a comparison between folk taxa (highly represented in the *Criollos* graded classification of lateral linkage) are a linguistic form commonly used by different cultural groups to order a nature domain [46–48]. While some authors consider it only as a pedagogical strategy [49], according to the methodology developed by us, we noted that it reflects a cognitive pattern and thus, implicitly a logic of classification based on family resemblances between more or less prototipycal folk taxa. According to the discussion developed by Newmaster et al. [4] in an “ethnographic reality, folk taxonomies appear to be classified more according to a complex web of resemblances” than to forming a neat hierarchy, so the term taxonomic hierarchies should be considered as a misnomer.

A common factor in a graded classification of lateral linkage is the broad and flexible use of comparisons that act as cognitive reference points. Both through direct comparisons, and through membership relations between generic and specific folk taxa, the *Criollos* use some folk taxon with great prototypical value to classify the domain. However, not all folk taxa have the same prototypical value [50]. Thus, we can say that *yateí* is the most prototypical folk taxon for the residents of our study. On the one hand, yateí was one of the folk taxa that reached higher values of PV and one of the greatest modelers of folk taxa groups observed in the PCA. On the other hand, yateí meets many of the features of a prototype, according to the Prototype Theory [6, 7]. *Yateí* is the most quoted folk taxon, the most used as cognitive reference point (in similarity judgments), the first learnt in childhood and the most commonly raised bee in domestic settings with aesthetic, recreational, and utilitarian purposes [51]. It is also the bee from which an excellent honey is extracted and which is used for food and medicinal purposes [34, 41, 52].

It is noteworthy that other distant cultures placed similar species of SB as prototypical in the domain. For example, the Pankararé of Bahia (Brasil) pointed *irapuá* (*carabozá* in this paper) as the folk taxon that defined the *irapua*’s group, which is determined by the aggressiveness of its members [44]. While among Mby’a of Misiones, *yateí* and *mandurí* are prototypical folk taxa representing two opposite forms: thin and small, and robust and large bees respectively [53]. In our observations among the *Criollos,* the *abeja* occupies a similar place to *mandurí* (genus *Melipona*) among Mby’a, since it represents the best prototypical folk taxon of those bees which are robust, large and have abdominal stripes. The results found in our work also agree with Bentley and Rodriguez [54] who found that Honduran farmers grouped two species of the genus *Melipona* together with *Apis mellifera* due to their size and morphological similarities.

The functional special-purpose classification was only recorded in the in-depth interview made to the key informant. We do not know if this form of classification is shared with other people, or if otherwise is part of an idiosyncratic knowledge of the informant. In this system category boundaries are more diffuse and may include other insects, different from stingless bees. The common denominator of a broader underlying domain is that all insects are honey producers. Since BL is one of the most knowledgeable informants in our study (mentioned and described 12 folk taxa), it is likely that this type of grouping was the result of expertise, similarly to that found by Boster and Johnson [55] among expert and novice fishermen. According to the authors, experts and novices differ not only in the amount but in the kind of information they control. However this hypothesis is beyond the scope of our work.

Binomiality and variability in nomenclature are part of the same processes but must be analyzed in different theoretical frameworks to improve their power of interpretation. This is, in a linguistic-cognitive framework and in the context of the processes of acquisition and transmission of knowledge, respectively. In turn binomiality allows us to explore socio-ecological meanings while variability in naming allows us to derive some hypothesis on the origin and meaning of local variability.

Regarding the first concern, Begossi and colleagues [21] found that the morphological variability of a particular group of organisms would condition the structure of names used. They argue that monomial, less explanatory names, are often used to describe individuals of groups with large morphological variability, whereas among groups with morphologically similar individuals, binomial names with mnemonic markers referring to distinctive features (e.g. color, size, etc.) allow to differentiate the folk taxa. Taking into account this assumption, we would expect to find a greater number of binomial names given that SB is a relatively uniform and small domain. It consists of 12 folk taxa and about 16 species, all belonging to a single tribe (Meliponini). Nevertheless, we reject this hypothesis because most commonly and stable (i.e., with higher agreement) names used here were monomials with the exception of *abeja de suelo*. Similarly, our observations do not support the hypothesis that suggests binomial names prevail in biological groups whose ecology is better known by local people [21]. It would only apply for certain over-differentiated folk taxon as *yateí,* where folk varieties are recognized and named with binomial names. However, often monomial names used by the *Criollos* are associated with higher agreement in the study population and therefore with better understanding of the SB domain. This is because people who have no knowledge about an aspect of their culture, a natural phenomenon, or other domain of knowledge tend to answer dissimilarly given the large range of possible responses. On the contrary the coincidence between people (agreement) is indicative of sharing cultural knowledge [29].

Our results agree with Tournon [24] who proposed that descriptive names such as binomial decrease as cultural knowledge, usages, and salience, increase. In the same line of argument Brown [22] proposed that less salient folk taxa require binomial names with mnemonic markers that function as memory aids. Thus, binomial names are associated with a principle of economy of memorization [22, 24]. For Brown [22], salience is indirectly given by the agreement on the use of a name, while overall salience is determined by cultural importance of the resource plus the salience of biological taxa (e.g. size, color, etc.). These ideas may explain the widespread use of monomial names in SB nomenclature, insofar as bees are highly salient organisms and considered important. They are commonly used in alimentation and medicine, and are important because they live in organized groups similarly to humans, thus standing as ready-made symbols of humanity [54, 56, 57]. In contrast to binomial names, monomial names are associated with a principle of economy of elocution that favors unitary terms [24].

Regarding our second concern, we interpret the use of names with low agreement, underrepresented but numerous, as descriptions or explanations. If we consider that meanings can change over time [19] these names may take root or replace other names later on. These underrepresented names are the result of associations, inferences and explanations of facts made by people who probably did not receive information about the cultural names -with high agreement- of some folk taxa, although they probably interacted with these bees at least once. Thus, the *Criollos* of Misiones practiced and tested their creative and logic capabilities creating new binomial names based on their prior knowledge of the domain (knowledge of bee ecology, morphology, properties of honey, etc.) and through relationships with other folk taxon acting as a reference point to SB groups and subgroups. Hence we find “unnamed bees”*,* “own creation” and “exceptions” within the repertoire of expressions used to refer to SBs.

From our point of view alternative naming and classification observed in our study can be better analyzed if we consider their agreement in the population. To the contrary, Tournon [24] indicates that agreement is not an absolute criterion to identify a real name of spontaneous description. He stated that faced with the same stimulus several people with “semantic knowledge” might give the same name based solely on what they see regardless of their “encyclopedic knowledge” of the organisms. We think that the mechanism suggested by Tournon [24] hardly allows the generation of names with high agreement, even when the appointed organism has salient features (eg. call a plant with many thorns “thorny”). To achieve consensus -a measure of agreement- requires that transmission networks of cultural based knowledge work properly [55]. This is particularly true when names of higher agreement are monomials that lack information (primary or unanalysable names) as those mostly used to name SBs in this study.

Two possible explanations about local naming variability (or alternative naming) reported are as follows: 1) the observed variation is not greater than the variability in the past. On one hand, it can be understood as an average variability that has been maintained over time, as a result of differences in expertise commonly observed within a domain in a given population [28, 55]. Moreover, variability can be understood as the product of a continuous process of change or substitution of names that has possibly originated from the change of meaning names suffer over time [19]. 2) The observed variation is greater than that of a past time. Variability results from a process of fragmentation of knowledge or a process of incomplete transmission of knowledge similar to that reported by Ohmagari and Berkes [58]. This process leans on the facts that culturally based knowledge, on the one hand, and biologic knowledge of nature on the other, can be acquired through different learning pathways [28, 59]. Here, we use the term “culturally based knowledge” to refer to the sum of semantic or theoretical knowledge (see [24, 60]) and to knowledge related to the practices and traditions of a cultural group and we use the term “biological knowledge of nature” to refer to local and traditional knowledge about the life history and ecology of individuals -populations or communities- and their environment.

We support more strongly the second hypothesis; we believe that the different expressions associated to the naming of bees (*own creation*, “exceptions”, “unnamed bees”) that are away from the higher agreement names, are tests which should be analyzed in relation to some modeler parameters of the local knowledge; these are the presence and abundance of SBs, the learning opportunity that depends on people’s time availability and the interest that people have on the resource [61]. In particular, the study area has undergone rapid change in terms of the appearance of its forests as well as in the socio-productive sphere. The reduction and fragmentation of forests due to colonization and development of industrial agriculture (tobacco, *yerba mate*, forest woods) is key to these changes [62]. These disturbances, as well as the time spent by people to “walk” the forest and interact with bees, affect the availability of bee colonies, especially those of genera susceptible to anthropogenic changes such as the genus *Melipona* (see [63]). In turn, the strong presence of shops and markets affect interests and promotes replacement of honey by manufactured products like refined sugar or remedies [42, 51]. This scenario produces what Ohmagari and Berkes [58] called a gap between the aging “expert generation” and the interest of the younger generation.

## Conclusions

Through the combined use of different theoretical and methodological approaches to study the classification of stingless bees we support the multidimensionality of folk classification systems. This confirms the specificity of local classification systems but also reflects the use of grouping strategies and mechanisms commonly observed in other groups such as the use of similarities judgments between more or less prototypical organisms. Up to the present this cognitive-psychological approach has not been used widely in ethnobiological studies and we believe it deserves more attention. Likewise we believe that “the emphasis on variability should not detract from a coherent cultural portrayal of the societies studied” [28] as it can give us valuable clues about silent processes such as those related to the acquisition and transmission of knowledge. Far from assuming the irretrievable loss of knowledge, it implies paying attention to its dynamic nature.
